# Malignant Hyperthermia in Slavonic cohort – clinical and genetic findings beyond standard diagnostics

**DOI:** 10.1186/s13023-026-04339-w

**Published:** 2026-03-31

**Authors:** Martina Klincová, Jana Zídková, Kamila Réblová, Dagmar Štěpánková, Ivana Schröderová, Renata Gaillyová, Tereza Bönischová, Natálie Havránková, Veronika Tomášková, Tereza Kramářová, Lenka Fajkusová, Petr Štourač

**Affiliations:** 1 Department of Paediatric Anaesthesiology and Intensive Care Medicine, University Hospital Brno and Faculty of Medicine, Masaryk University, Brno, Czech Republic; 2 Academic Centre for Malignant Hyperthermia of Masaryk University, Brno, Czech Republic; 3 Department of Simulation Medicine, Faculty of Medicine, Masaryk University, Brno, Czech Republic; 4 Centre of Molecular Biology and Genetics, Department of Internal Medicine, Haematology and Oncology, University Hospital Brno and Faculty of Medicine, Masaryk University, Brno, Czech Republic; 5 Institute of Medical Genetics and Genomics, University Hospital Brno, Brno, Czech Republic and Faculty of Medicine, Masaryk University, Brno, Czech Republic; 6 Central European Institute of Technology, Masaryk University, Brno, Czech Republic; 7 Department of Anaesthesiology and Intensive Care, St Anne’s University Hospital and Faculty of Medicine, Masaryk University, Brno, Czech Republic; 8 National Centre for Biomolecular Research, Faculty of Science, Masaryk University, Brno, Czech Republic

**Keywords:** Malignant hyperthermia, IVCT, RYR1, VUS

## Abstract

**Background:**

Malignant Hyperthermia (MH) is a rare, hereditary, life-threatening pharmacogenetic disorder. The genetic background of MH was identified only in approximately half of MH-susceptible (MHS) patients, differing between geographical areas. Variants recognised as being sufficiently functionally characterised to be used in diagnostics for MH are on the “European Malignant Hyperthermia Group list of diagnostic variants”. However, the list may be a bit biased in the sense of rare variants in favour of better-described and published MHS cohorts. Also, the understanding of MH genetics is incomplete, with no variant identified in a significant number of cases. Aimed to investigate the clinical features, detected gene variants, and potential genotype-phenotype correlations in Slavonic patients with MH.

**Results:**

Retrospective analysis of clinical data and molecular genetic characteristics was conducted on patients referred for possible MH during 20 + years of diagnostics at the Academic Centre for Malignant Hyperthermia of Masaryk University, Brno, Czech Republic. MH susceptibility was confirmed in 94 families, containing 254 individuals. 79% of our MHS probands were diagnosed based on MH-related adverse anaesthesia complications in personal/family history. Pathogenic/Likely-pathogenic MH variants in the *RYR1* gene were detected in 48% of MHS families. In 18% of MHS patients, 14 variants of unclear significance (VUS) were detected. Three of these VUS were detected in more than one unrelated family - c.1589G>A p.(Arg530His), c.1598G>A p.(Arg533His), and c.6742C>T p.(Arg2248Cys). We detected five families with one example of genotype-phenotype discordance. Still, in 34% of MHS probands, no variants were detected. Expanded diagnostics with neuromuscular panels or exome sequencing did not provide any additional benefit. We also presented the list of five patients with detected VUS and MH-negative In Vitro Contracture Test (IVCT).

**Conclusions:**

This study presents the largest Slavonic cohort of patients investigated for risk of MH. Nearly half of the detected *RYR1* variants were VUS, though most appear as likely pathogenic for MH in our cohort and could be considered for a change in pathogenicity criteria. Still, over a third of MHS patients lack a confirmed genetic basis and may benefit from future advances in molecular genetic diagnostics and new scientific knowledge.

**Registration:**

This is a retrospective observational national cohort study reported according to STROBE guidelines (https://www.strobe-statement.org/index.php?id=strobe-home). The study was registered on 05/09/2021 at ClinicalTrials.gov (ID: NCT05036148).

**Supplementary Information:**

The online version contains supplementary material available at 10.1186/s13023-026-04339-w.

## Background

Malignant Hyperthermia (MH) is a rare, hereditary, life-threatening pharmacogenetic disorder triggered by some commonly used anaesthetics [1]. Pathophysiologically, severe dysregulation of skeletal muscle Ca2 + homeostasis results in the clinical features of an MH reaction under anaesthesia. Based on current knowledge, a causal relationship has been demonstrated between MH and genetic variants at the level of excitation-contraction (E-C) coupling, specifically in the ryanodine receptor RyR1 on the sarcoplasmic reticulum membrane (*RYR1* gene), the voltage-gated calcium channel Cav1.1 located in the T-tubular membrane of myocytes (*CACNA1S* gene), and the protein encoded by the *STAC3* gene [2–4].

However, the phenotypes associated with genetic defects predisposing to MH are not confined to reactions to volatile anaesthetics and imply a common or overlapping pathophysiology with myopathies and exertional rhabdomyolysis [5]. The incidence of MH crisis is approximately 1:10,000–1:250,000 anaesthesia depending on population and anaesthetic management [1], but the combined prevalence of all MH pathogenic/likely pathogenic variants in total is much higher 1:2750 [5], according to newer data even 1:605 [6].

Molecular genetic testing is now a standard part of MH diagnostics, but it has its limitations and hurdles. *RYR1* has been established as the major gene implicated in MH; variants are gain-of-function, making mutant RyR1 channels more sensitive to activation [7]. The majority of *RYR1* variants found in databases are rare. The correct interpretation of detected variants is very important, and sometimes, without In Vitro Contracture Test (IVCT) results even impossible. Variants recognised as being sufficiently functionally characterised to be used in diagnostic genetic testing for MH are on the “European Malignant Hyperthermia Group (EMHG) list of diagnostic variants” [8], but the list may be a bit biased in the sense of rare variants in favour of better-described and published MH-susceptible (MHS) cohorts. The EMHG has modified the American College of Medical Genetics and Genomics (ACMG) classification [9] to better fit the MH-susceptibility and proposed the EMHG Scoring Matrix [10]. However, the classification of *RYR1* variants is complicated by variable expressivity, reduced penetrance, and high allelic heterogeneity [11]. The genetic predisposition to MH was initially thought to follow an autosomal dominant inheritance pattern in *RYR1* and *CACNA1S* genes [1], but nowadays it is speculated that the penetrance of a genetic trait is incomplete, and the genetic defect either requires additional factors for the phenotype to occur, or other factors may prevent the occurrence of the phenotype [5].

Overall, our understanding of MH genetics remains incomplete, with no variant identified in a significant number of cases and considerable phenotype diversity [12]. The genetic background of MH was identified only in approximately 40–60% of MHS patients, differing between geographical areas [13–18]. Current scientific research aims to further reveal the underlying genetic cause of MH. To contribute, we decided to publish data describing the clinical and molecular characteristics of our MHS Slavonic cohort of patients. In addition, we aimed to deepen our knowledge about the aetiopathogenesis of MH by performing extended genetic diagnostics in our MHS group of patients with yet unidentified genetic basis. Finally, we also present variants of uncertain significance (VUS) connected with a negative In Vitro Contracture Test (IVCT).

## Methods

### Ethics approval

All patients or their parents gave their informed consent for the use of their clinical and laboratory data for further analysis and MH-related research including publication (approved by the Hospital Ethical Board, Brno, Czech Republic).

### Registration

This is a retrospective observational national cohort study reported according to STROBE guidelines (https://www.strobe-statement.org/index.php?id=strobe-home). The study was registered on 05/09/2021 at ClinicalTrials. gov (ID: NCT05036148).

### Setting

We screened all probands referred to the Academic Centre for Malignant Hyperthermia of Masaryk University (ACMH MU) in Brno, Czech Republic, which has adopted the EMHG diagnostic guidelines and is responsible for MH diagnostics in our region since 2002. The data collection was conducted from September 2021 to the end of September 2024.

### MH diagnostic process

Probands were examined strictly according to the diagnostic recommendations of the EMHG valid at the time of reporting by molecular genetic testing and/or muscle IVCT [19, 20]. Formerly, the IVCT used to be the first diagnostic step and genetic testing was performed afterwards. Nowadays, the MH diagnostic process is usually initiated with genetic testing. If a pathogenic/likely pathogenic (PV/LP) variant is detected, the patient is diagnosed as MH susceptible (MHS) based on genetic testing only; otherwise, the IVCT must be performed to complete the MH diagnostic process and to confirm or exclude the MH susceptibility.

### Participants

Each proband represents one unrelated family. All probands with a completed diagnostic process were eligible for analysis. Excluded were proband referrals, where the MH diagnostic process was not recommended or, for various reasons, was not completed. In a group of the MHS probands, we performed an analysis of clinical issues accountable for each proband´s referral (reason of referral, adverse anaesthesia complication in personal or family history, administration of dantrolene); IVCT result (MHS_hc_/MHS_h_/MHS_c_ = MH susceptible IVCTs with abnormal reaction to both halothane and caffeine/only to halothane/only to caffeine); genetic findings; number of relatives with positive IVCT; number of relatives with the same variant (if applicable). In the MH-negative (MHN) group, in the sense of this article, we specifically listed only the detected VUS connected with MHN IVCT.

### Detection of genetic variants

For all reported probands after 2020, the primary genetic diagnosis was conducted via the Next-generation Sequencing (NGS). For all MHS probands without detected diagnostic variants before 2020, a reanalysis with the NGS panel was completed. To identify sequence variants in genes associated with MH, we used the capture method KAPA HyperChoice (Roche) followed by NGS on the NextSeq 500 (Illumina) of a panel of genes associated with neuromuscular diseases (www.musclegenetable.fr). Custom capture probes were used for routine diagnostics, in the case of selected families we used exome probes (KAPA HyperExome, Roche). The individual DNA sequence reads were aligned to the published human genome reference hg19/GRCh37 and variants were called using the software CLC Genomics Workbench (QIAGEN). Identified variants were evaluated using the ACMG guidelines [9], the EMHG guidelines [8, 10], the VarSome variant classifier (https://varsome.com) and the HGMD (Human Gene Mutation Database). Pathogenicity predictions based on REVEL and metaRNN scores were also included. All PV/LP variants were confirmed by PCR and Sanger sequencing using the BigDye Terminator Cycle Sequencing Kit (Applied Biosystems) on the ABI 3130xl Genetic Analyzer (Applied Biosystems). Bioinformatics reanalysis was conducted on previously performed genetic exams not initially part of routine diagnostics. For whole exome sequencing (WES), we analysed families with at least 2 members with positive IVCT and one member with negative IVCT. Detected variants with an allele frequency greater than 1% within The Genome Aggregation Database (gnomAD v4.1) were excluded from further analysis. Intronic and synonymous variants, and variants categorized as benign or likely benign in the database ClinVar were also eliminated from consideration. The remaining variants were compared between members with positive and negative IVCT, considering the type of variant, in silico analysis of missense and splicing variants, and gene’s function and expression. Whole genome sequencing (WGS) was not performed in our dataset, as it is not currently part of routine clinical diagnostics for MH in our region.

### Statistical analyses

The data were reported descriptively using Microsoft 365 (Microsoft Corporation).

## Results

As of the end of September 2024, our database contains 350 probands reported to the ACMH MU, 320 from the Czech Republic, 29 from Slovakia and one from Poland. Each proband is a representative of one unrelated family. MH diagnostics were completed in 184 probands. MH-susceptibility was confirmed in a total of 94 probands/families, containing 254 individuals. 74 (79%) of our MHS probands were diagnosed based on MH-related adverse anaesthesia complications in personal/family history; there was only a slightly decreasing trend in the frequency of anaesthesia-related referrals among groups with diagnostic variant, VUS, or without any variant, 82%, 76% and 75%, respectively.

Pathogenic/likely pathogenic (PV/LP) MH variants in the *RYR1* gene were detected in 45 (48%) of our MHS patients; 14 different PV in 39 unrelated families, and 3 LP variants in 6 unrelated families (Table 1). The most prevalent pathogenic variants detected in our MHS patient cohort were: c.1840C>T p.(Arg614Cys) (8 families), c.6617C>T p.(Thr2206Met) (8 families), and c.6502G>A p.(Val2168Met) (5 families).

**Table 1:** Detected *RYR1* pathogenic/likely pathogenic variants from EMHG list [8] of diagnostic variants in patients with malignant hyperthermia

RYR1 variant (NM_000540.2)	EMHG list of diagnostic variants	Number of probands	Proband ID	Reason of referral	Adverse anaesthesia complication in personal or family history	Administration of dantrolene	IVCT	Relatives with positive IVCT	Relatives with the same variant
c.529C>T p.(Arg177Cys)	Likely pathogenic	1	p1	Incidental finding in genetic testing	unclear	unknown	not performed	no	0
c.8026C>T p.(Arg2676Trp)	Likely pathogenic	4	p2	MH crisis - typical	yes	yes	MHS_hc_	1#	no
			p3	Trismus, rhabdomyolysis	yes	no	MHS_hc_	6#	3
			p4	HyperCK, myopathy	no	n/a	not performed	no	no
			p5	MH crisis - typical	yes	yes	not performed	no	no
c.12533G>T p.(Gly4178Val)	Likely pathogenic	1	p6	MH crisis - typical	yes	yes	MHS_hc_	2#	1
c.487C>T p.(Arg163Cys)	Pathogenic	2	p7	MH death in family	yes	no	not performed	no	1
			p8	Rhabdomyolysis, myopathy	no	n/a	not performed	no	2
c.488G>T p.(Arg163Leu)	Pathogenic	1	p9	MH crisis - typical	yes	yes	MHS_hc_	no	4
c.1840C>T p.(Arg614Cys)	Pathogenic	8	p10	MH crisis - typical	yes	yes	MHS_hc_	no	no
			p11	MH crisis - typical	yes	unknown	MHS_hc_	2#	4
			p12	MH crisis - typical	yes	unknown	MHS_hc_	no	no
			p13	MH death in family	yes	no	not performed	no	10
			p14	MH death in family	yes	no	MHS_hc_	4	14
			p15	MH crisis - postoperative rhabdomyolysis	yes	no	MHS_hc_	no	no
			p16	Incidental finding in genetic testing	no	n/a	not performed	no	no
			p17	Incidental finding in genetic testing	no	n/a	not performed	no	1
c.1841G>T p.(Arg614Leu)	Pathogenic	1	p18	MH crisis - typical	yes	yes	MHS_hc_	no	5
c.6488G>A p.(Arg2163His)	Pathogenic	1	p19	MH crisis - typical	yes	unknown	MHS_hc_	no	no
c.6502G>A p.(Val2168Met)	Pathogenic	5	p20	MH crisis - typical	yes	yes	not performed	no	5
			p21	MH crisis - typical	yes	no	MHS_hc_	no	2
			p22	MH crisis - typical	yes	unknown	MHS_hc_	no	4
			p23	MH death in family	yes	no	MHS_hc_	no	1
			p24	Trismus, rhabdomyolysis	yes	no	not performed	no	1
c.6617C>T p.(Thr2206Met)	Pathogenic	8	p25	MH crisis - typical	yes	unknown	MHS_hc_	no	3
			p26	MH crisis - typical	yes	unknown	MHS_hc_	no	10
			p27	Trismus, rhabdomyolysis	yes	no	MHS_hc_	no	2
			p28	Postoperative rhabdomyolysis	yes	no	MHS_hc_	no	13
			p29	HyperCK	no	n/a	not performed	no	2
			p30	MH crisis - typical, CCD	yes	yes	not performed	no	1
			p31	Myopathy	no	n/a	not performed	no	1
			p32	Myopathy	no	n/a	not performed	no	no
c.7042_7044del p.(Glu2348del)	Pathogenic	1	p33	Postoperative hyperthermia	yes	no	MHS_hc_	3	3
c.7048G>A p.(Ala2350Thr)	Pathogenic	2	p34	MH crisis - typical	yes	yes	MHS_hc_	no	no
			p35	Trismus, rhabdomyolysis	yes	no	MHS_hc_	no	1
c.7063C>T p.(Arg2355Trp)	Pathogenic	1	p36	MH crisis - typical	yes	yes	not performed	no	no
c.7300G>A p.(Gly2434Arg)	Pathogenic	1	p37	MH death in family	yes	no	MHS_hc_	no	3
c.7361G>A p.(Arg2454His)	Pathogenic	2	p38	MH death in family	yes	no	MHS_hc_	no	1
			p39	MH crisis - typical	yes	yes	not performed	no	1
c.7373G>A p.(Arg2458His)	Pathogenic	4	p40	MH crisis in family	yes	no	MHS_hc_	2	10
			p41	Postoperative hyperthermia	yes	no	MHS_hc_	no	3
			p42	MH death in family	yes	yes	not performed	no	1
			p43	MH crisis - typical	yes	unknown	MHS_hc_	no	1
c.7523G>A p.(Arg2508His)	Pathogenic	2	p44	MH crisis - typical	yes	yes	MHS_hc_	no	1
			p45	MH crisis - typical	yes	yes	not performed	no	no

MH = Malignant hyperthermia, *RYR1* = Ryanodine receptor isoform 1 gene, IVCT = In Vitro Contracture Test; MHS_hc_/MHS_h_/MHS_c_ = MH susceptible IVCTs with abnormal reaction to both halothane and caffeine/only to halothane/only to caffeine, CK = Creatine kinase, CCD = Central Core Disease; #Discordance in family - one familial variant negative person had positive IVCT

In 17 (18%) unrelated MHS patients, 14 VUS for MH were detected in the *RYR1* gene (Table 2). Three of these VUS were detected in more than one unrelated family - c.1589G>A p.(Arg530His), c.1598G>A p.(Arg533His), and c.6742C>T p.(Arg2248Cys). The REVEL and the metaRNN scores in this VUS MHS group varied from 0.676 to 0.974; from 0.186 to 0.9902, respectively. The identified variants, together with their interpretations based on the EMHG [8] and the important clinical data, are listed in Tables 1 and 2; the full data list with more evaluation criteria is attached as an E-supplement 1. Variants associated with MH in the *CACNA1S* and *STAC3* genes were not detected in our MHS cohort.

**Table 2:** Detected *RYR1* variants of uncertain significance in patients with malignant hyperthermia

RYR1 variant (NM_000540.2	EMHG Scoring Matrix	Number of probands	Proband ID	Reason of referral	Adverse anaesthesia complication in personal or family history	Administration of dantrolene	IVCT	Relatives with positive IVCT	Relatives with the same variant
c.49G>T p.(Asp17Tyr)	PPb	1	p46	Myopathy	no	n/a	MHS_hc_	no	no
c.178G>A p.(Asp60Asn)	PPb PPc	1	p47	Postoperative hyperthermia	yes	no	MHS_hc_	1	1
c.946C>T p.(Arg316Cys)	PPb PPc	1	p48	MH crisis in family	yes	no	MHS_hc_	no	no
c.1589G>A p.(Arg530His)	PPb PPc	2	p49	Trismus	yes	yes	MHS_h_	no	1
			p50	MH crisis - typical	yes	yes	MHS_hc_	no	no
c.1598G>A p.(Arg533His)	PPb PPc	2	p51	Incidental finding in genetic testing	no	n/a	not performed	no	2
			p52	MH crisis	yes	unknown	MHS_hc_	2#	1
c.1762C>T p.(Leu588Phe)	PPb PPc	1	p53	MH crisis, CCD	yes	no	MHS_h_	no	no
c.6385G>A p.(Asp2129Asn)	PPb PPc	1	p54	MH crisis	yes	no	MHS_h_	1	1
c.6742C>T p.(Arg2248Cys)	PPb PPc	2	p55	MH crisis	yes	unknown	MHS_h_	1	1
			p56	MH crisis in family	yes	no	MHS_h_	no	no
c.6863T>C p.(Leu2288Ser)	PPb PPc	1	p57	MH crisis	yes	unknown	MHS_hc_	no	no
c.7035C>A p.(Ser2345Arg)	PPb PPc	1	p58	MH death in family	yes	no	MHS_hc_	1	1
c.7087T>C p.(Cys2363Arg)	PPb PPc	1	p59	MH death in family	yes	no	MHS_hc_	no	no
c.7268T>A p.(Met2423Lys)*	PPb	1	p60	Myopathy	no	n/a	MHS_h_	no	no
c.10648C>T p.(Arg3550Trp)	BPa	1	p61	Incidental finding in genetic testing	no	n/a	MHS_h_	no	2
c.7210G>A p.(Glu2404Lys)	PPb PPc	1	p62	Postoperative rhabdomyolysis	yes	no	MHS_hc_	1	1

MH = Malignant hyperthermia, *RYR1* = Ryanodine receptor isoform 1 gene, IVCT = In Vitro Contracture Test; MHS_hc_/MHS_h_/MHS_c_ = MH susceptible IVCTs with abnormal reaction to both halothane and caffeine/only to halothane/only to caffeine, CK = Creatine kinase, CCD = Central Core Disease; #Discordance in family - one familial variant negative person had positive IVCT; * inconsistency – this variant related to both positive (MHS_h_) and negative IVCT (see also Table 4)

The genotype-phenotype discordances, where one family member was lacking the familial variant but had a positive IVCT, were detected in five MHS families, in four out of 45 families carrying a PV/LP *RYR1* variant and in one family in the VUS MHS group. The percentage of familial variant negative and IVCT positive individuals from all 254 MHS individuals was 2%.

**Fig. 1:**
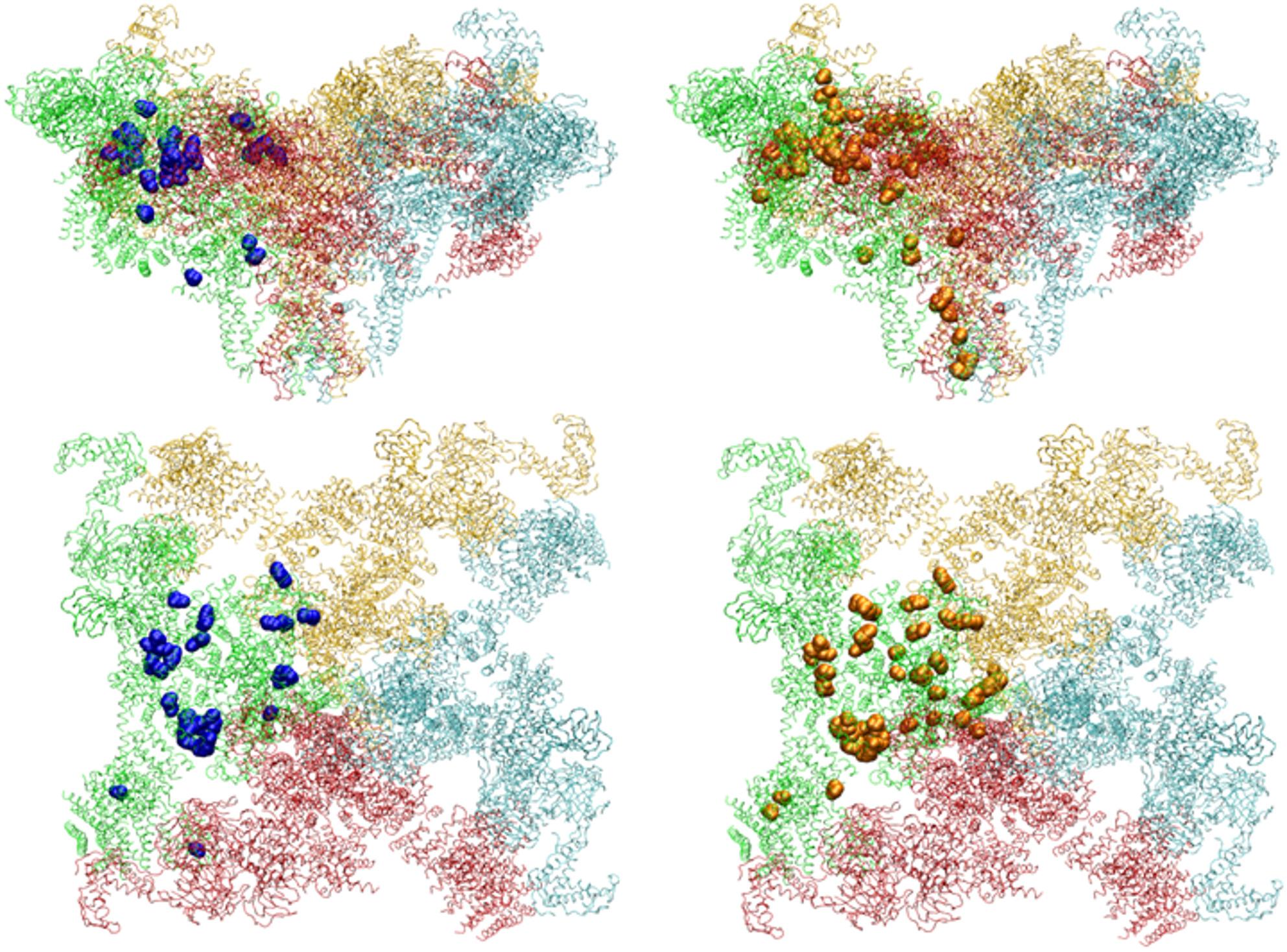
Visualisation of our and known pathogenic/likely pathogenic MH variants in the 3D RyR1 structure. Legend: our variants (blue), known pathogenic/likely pathogenic MH variants (https://www.emhg.org/diagnostic-mutations) (orange) in the 3D RyR1 structure (5gky.pdb, closed form), top and side views

We analysed our RYR1 variants’ properties and mapped them onto the 3D RyR1 structure, revealing that both our and known MH variants are scattered throughout (Fig. 1). As shown in E-Supplement 2, most are linked to a charge change, including 81% of our MH variants and 69% of known MH variants. In addition, some variants are coupled with gain or loss of phosphorylation (it concerns serine, tyrosine and threonine substitutions), which can impact changes in protein interactions. Rest variants (not coupled with change of charge or phosphorylation) are often very conservative substitutions, e.g. p.(Leu588Phe), p.(Phe539Leu), p.(Val4234Leu), p.(Leu4838Val), p.(Val4849Ile), which may fine-tune the activity of the channel.

Still, in 32 (34%) MHS probands, no variants were detected. Table 3 describes this group of probands based on referral reason and IVCT results. NGS analysis of other neuromuscular genes revealed no variants of concern. For the WES analysis, we selected four families with known MH susceptibility, three families were referred for adverse anaesthesia complications and one for myopathy. A total of 21 patients were analysed: 14 with positive and 7 with negative IVCT results. No potentially pathogenic variants or novel causative genes associated with MH susceptibility were identified in this analysis.

**Table 3:** MH-susceptible probands without any detected variant

	Referral reasons					In total
	Adverse anaesthesia complication in personal or family history		Myopathy	Rhabdomyolysis, hyperCK	Other	
	with dantrolene	without dantrolene				
Number of probands	5	19	1	1	6	32
IVCT result	1x MHS_hc_	2x MHS_hc_	1x MHS_hc_	-	-	4
	2x MHS_h_	14x MHS_h_	-	1x MHS_h_	4x MHS_h_	21
	2x MHS_c_	3x MHS_c_	-	-	2x MHS_c_	7
Families with > 1 positive IVCT	2	3	1	0	2	8

MH = Malignant hyperthermia, IVCT = In Vitro Contracture Test; MHS_hc_/MHS_h_/MHS_c_ = MH susceptible IVCTs with abnormal reaction to both halothane and caffeine/only to halothane/only to caffeine

Finally, we also present the list of four patients with VUS in the *RYR1* gene and one patient with a combination of variants in genes *CACNA1S* c.1856T>G p.(Met619Arg) and *CASQ1* c.757G>A p.(Val253Ile) from the group of our 90 MHN probands connected with negative IVCT (Table 4).

**Table 4:** Variants of uncertain significance connected with MHN IVCT

Proband ID	Reason for referral	Detected VUS	IVCT
n1	Myopathy	*RYR1* c.2198G>A p.(Gly733Glu)	MHN
n2	Incidental finding in genetic testing	*RYR1* c.7904A>T p.(Glu2635Val)	MHN
n3	Myopathy	*CACNA1S* c.1856T>G p.(Met619Arg), *CASQ1* c.757G>A p.(Val253Ile)	MHN
n4	Incidental finding in genetic testing	*RYR1* c.7268T>A p.(Met2423Lys)*	MHN
n5	Incidental finding in genetic testing	*RYR1* c.3257G>A (p.Arg1086His)	MHN

MH = Malignant hyperthermia, IVCT = In Vitro Contracture Test, MHN = Malignant hyperthermia negative; *RYR1* NM_000540.2, *CACNA1S* NM_000069.2, *CASQ1* NM_001231.4; * inconsistency – this variant related to both negative and positive (MHS_h_) IVCT (see also Table 2)

## Discussion

This report presents a unique characterisation of a malignant hyperthermia susceptible (MHS) cohort from the Slavonic part of Europe, previously described only marginally [18]. With ongoing advances in genetic diagnostics, integrating genetic findings with clinical correlations is essential for refining pathogenicity assessment of rare variants and enabling less invasive MH diagnostics. Despite the increase of referrals with incidental findings in genetic testing in the NGS era [21], the majority of our MHS probands were still diagnosed based on personal or family anaesthesia complications.

Compared to 2022, when pathogenic *RYR1* variants were detected only in 44% of our MHS families [18], nowadays with 48% is the prevalence is only slightly higher. As a side effect of constant reassessing of variants and introduction of the EMHG Scoring Matrix [10], the *RYR1* variants c.1589G>A p.(Arg530His) and c.1598G>A p.(Arg533His), which at the beginning of 2022 still were on the EMHG list of diagnostic variants, were later excluded [8]. However, there are two unrelated families in our MHS cohort for each variant. The variant c.1589G>A p.(Arg530His) was detected in two of our probands with MH crises (IDs p49 and p50) previously also detected in British, Swiss, Italian and Japan MHS cohort [13, 17, 22–24], and according to ACMG and ClinVar, the variant is still described as likely pathogenic. The variant c.1598G>A p.(Arg533His) was also detected in two probands (IDs p51 and p52), one with MH crisis and MHS_hc_ IVCT. In the second proband, the variant was detected incidentally, and IVCT was not performed as it was still classified as diagnostic by the EMHG at that time. This variant was previously detected also in British and Japan MHS cohort [13, 25].

From this perspective, the most important contribution of this study is presenting the group of MHS patients with VUS and their clinical correlations (Table 2). Overall, 11 of our VUS can be considered for change in pathogenicity classification (E-supplement 1, PPb PPc according to the EMHG Scoring Matrix) [10]. Unfortunately, with our relatively small MHS cohort and gnomAD with “only” 622 thousand individuals from the control low-risk European population, none of our VUS meets the strict criteria for a variant to be described as LP and added to the EMHG list of diagnostic variants [8, 10]. Broader data sharing among MH centres may allow these criteria to be met in the future. Expanding the EMHG list of diagnostic variants would have a great impact on patients investigated for risk of MH, as when following the EMHG MH diagnostics guidelines, the MHS status could be proved based on the detection of the familial variant in blood, and the IVCT would be needed only for relatives without the detected variant to rule out the MH risk.

For example, variant c.7035C>A p.(Ser2345Arg) was detected in the family (ID p58), where the brother of our proband died at the age of 15 during testicle retention surgery due to MH crisis. The variant was detected in two relatives with MHS_hc_ IVCT and was not detected in the other two relatives with MHN IVCT; it has high metaRNN and REVEL scores; and causes both change of charge and possibly phosphorylation. According to ACMG criteria and ClinGen expert panel, this variant is likely pathogenic but has not been included yet in the EMHG list of diagnostic variants. This variant was also previously described in one Dutch and one Italian family with MHS and myopathy [26, 27]. Similarly, c.6742C>T p.(Arg2248Cys), identified in two unrelated probands with MH crises in our cohort, has also been reported in Italian and British MHS probands [28, 29].

Certainly, not every detected VUS will lead to likely pathogenicity. Even in our MHS VUS group, the variant c.10648C>T p.(Arg3550Trp) seems to be leaning more toward the benign direction - the very low metaRNN score of 0.186, missing correlation with clinical MH reaction (ID p61), In this case, IVCT performed at the family’s request yielded an MHS_h_ result. The interpretation of the variant c.7268T>A p.(Met2423Lys) is also very problematic, as it is pathogenic for myopathy, but VUS for MH. Our proband (ID p60) was referred because of myopathy, her IVCT was MHS_h_. However, she also had another *RYR1* variant causing deletion with frameshift c.2505del p.(Pro836Leufs*48), and the positive IVCT result might be due to the compounding effect of both of them. Moreover, this variant was also detected in proband ID n4 (Table 4) with MHN IVCT. The variant appears relatively common in our population and has been reported repeatedly in the literature [30–33]. The EMHG is planning to introduce a list of “Variants unlikely to cause malignant hyperthermia”, which would be extremely helpful for clinical counselling. All MH centres are invited to report more details about VUS and IVCT results (https://www.emhg.org/ryr1-unknown-significance). For these reasons, we have also listed the VUS with negative IVCT (Table 4).

The genotype-phenotype discordance detected in five families, where a familial variant negative person had positive IVCT, was detected in 2% of our MHS individuals. Similar discordance was previously described in other cohorts as well [13, 34–37]. Published data could support the statements in the introduction that very few *RYR1* variants are sufficiently penetrant to consistently cause MH susceptibility in the absence of other genetic risk factors [5]. Avoiding false MHN diagnoses based solely on molecular testing without negative IVCT is therefore critical, a point emphasised in current EMHG guidelines [19, 20].

Interestingly, in the MHS group with diagnostic variants, all IVCTs performed were MHS_hc_. On the contrary, the MHS_hc_ IVCTs were decreasingly less common among groups with VUS or without any detected variant, responsible for 53%, respectively only 13% MH susceptibility. Similar differences were previously described [38, 39], but the underlying pathology behind the observed phenotypic variation remains to be elucidated [12]. A recent study links MH susceptibility to dysregulated gene expression in mitochondrial bioenergetics, potentially explaining phenotypic variability [12]. The high proportion of IVCTs where the muscle sample reacted to only one substance, either to halothane (MHS_h_) or to caffeine (MHS_c_), may also raise the question of false positive borderline results, even though the IVCT sensitivity and specificity should be high, 99.0% and 93.6% respectively [40]. From a patient safety perspective, most of the probands were referred because of adverse anaesthesia complications in personal or family history, and an IVCT false positivity result will always be much safer for patients, as only trigger free anaesthesia will be performed in future.

This study is limited by a relatively small patient cohort and challenges in applying our data to other Slavonic populations (Polish, Ukrainian, Balkan, etc.). A major limitation may be our decision not to exclude patients without the MH anaesthesia phenotype from this report. Our rationale was that if a PV/LP MH variant is detected even incidentally, the patient is at risk of developing MH if trigger-free anaesthesia is not provided, and the fact that MH has not occurred yet, may only be due to a lack of exposure. The situation is even more complicated and complex with VUS for MH (we did not report the VUS without the IVCT results, though). Although not knowing the precise clinical phenotype complicates academic discussions, it is outweighed by patient safety. Reporting incidental findings is also problematic; ACMG recommends reporting only pathogenic or likely pathogenic variants [9, 41], but this is complicated for PV/LP variants for myopathy, often VUS for MH. Clinically, all RYR1-related myopathic patients are considered at risk for MH unless a negative IVCT proves otherwise. Variant classifications in this study are based on the EMHG diagnostic variant list available at the time of data analysis; subsequent updates to the EMHG list occurred during the review process.

NGS enables rapid, cost-effective sequencing of all protein-coding regions in the human genome. However, WES produces numerous variants across multiple genes, making their relevance to a specific phenotype hard to determine. Nowadays, the use of NGS-based targeted sequencing of a restricted panel of genes associated with a disease phenotype seems to be a more practical approach [5]. Unfortunately, the neuromuscular NGS panel in our cohort did not identify additional MH-related variants in E-C coupling genes beyond those already known. Nor did the WES. Our study confirmed the findings of previous studies with no discovery of novel MH candidate genes [29, 42–44]. Nonetheless, the research is ongoing but requires international data sharing, especially among related regions and ethnicities.

## Conclusion

This study presented the largest Slavonic MHS cohort from 20+ years of diagnostics. Nearly half of the detected *RYR1* variants were VUS, though most appear as likely pathogenic for MH in our cohort and could be considered for a change in pathogenicity criteria. However, over a third of MHS patients lack a confirmed genetic basis and may benefit from future advances.

## Supplementary Information

Below is the link to the electronic supplementary material.

Supplementary Material 1: E-supplement 1: All detected variants in the *RYR1* gene in our MH-susceptible cohort with detailed interpretation based on several criteria and clinical details. Attached Excel file. Legend: rs number = Reference SNP (Single Nucleotide Polymorphism) cluster ID; NFE European (non-Finnish); REVEL and metaRNN are pathogenicity prediction scores for human nonsynonymous single nucleotide variantsSNVs; ranging from 0 to 1, with higher scores reflecting the greater likelihood that the variant is disease-causing; HGMD (Human Gene Mutation Database); DM disease-causing mutation, DM? - likely disease-causing mutation; M male, F female; n/a not available

Supplementary Material 2: E-supplement 2: Survey of our and known MH variants (https://www.emhg.org/diagnostic-mutations). Identical variants in both groups are highlighted in red

## Data Availability

Data are available upon reasonable request.

## Abbreviations

ACMG: American college of medical genetics and genomics
ACMH MU: Academic centre for malignant hyperthermia of masaryk university
CACNA1S: Calcium channel, voltage-dependent, l type, alpha 1s subunit
CASQ1: Calsequestrin 1
CCD: Central core disease
CK: Creatine kinase
ClinVar: ClinVar is a freely accessible, public archive of reports of the relationships among human variations and phenotypes hosted by the national center for biotechnology information (NCBI) and funded by intramural national institutes of health (NIH) funding
ClinGen: Clinical genome resource. ClinGen is a national institutes of health (NIH)-funded resource dedicated to building an authoritative central resource that defines the clinical relevance of genes and variants for use in precision medicine and research
DNA: Deoxyribonucleic acid
EMHG: European malignant hyperthermia group
GnomAD: Genome aggregation database
HGMD: Human gene mutation database
IVCT: In Vitro contracture test
LP: Likely pathogenic variant
MH: Malignant hyperthermia
MHN: MH negative
MHS: MH susceptible
MHS_c_: a result of positive (MHS) IVCT, where the muscle reacts abnormally only to caffeine
MHS_h_: a result of positive (MHS) IVCT, where the muscle reacts abnormally only to halothane
MHS_hc_: a result of positive (MHS) IVCT, where the muscle reacts abnormally to both halothane and caffeine
NGS: Next-generation sequencing
PV: Pathogenic variant
RYR1: Ryanodine receptor isoform 1
STAC3: The protein encoded by this gene is a component of the excitation-contraction coupling machinery of muscles. This protein is a member of the Stac gene family and contains an N-terminal cysteine-rich domain and two SH3 domains
VUS: Variant of uncertain significance
WES: Whole exome sequencing
WGS: Whole genome sequencing
