# Supplementary material for: Malignant Hyperthermia in Slavonic cohort – clinical and genetic findings beyond standard diagnostics
Source: Orphanet J Rare Dis. 2026 Mar 31;21:192. doi: 10.1186/s13023-026-04339-w (PMC13159318; doi:10.1186/s13023-026-04339-w)
Supplement: Supplementary file 2 — Supplementary Material 2: E-supplement 2: Survey of our and known MH variants (https://www.emhg.org/diagnostic-mutations). Identical variants in both groups are highlighted in red [file 13023_2026_4339_MOESM2_ESM.docx]

| **Our MH variants** | **Classification ACMG** | **Change of charge** | **Possible phosphorylation** | **Known MH**  **variants** | **Classification EMHG** |
| --- | --- | --- | --- | --- | --- |
|  |  | yes |  | p.(Leu13Arg) | Pathogenic |
| c.49G>T p.(Asp17Tyr) | VUS | yes | yes |  |  |
|  |  | yes |  | p.(Cys35Arg) | Pathogenic |
|  |  | yes |  | p.(Arg44Cys) | Pathogenic |
| c.178G>A p.(Asp60Asn) | VUS | yes |  |  |  |
| c.487C>T p.(Arg163Cys) | Pathogenic | yes |  | p.(Arg163Cys) | Pathogenic |
| c.488G>T p.(Arg163Leu) | Pathogenic | yes |  | p.(Arg163Leu) | Pathogenic |
| c.529C>T p.(Arg177Cys) | Pathogenic | yes |  | p.(Arg177Cys) | Likely pathogenic |
|  |  | yes |  | p.(Gly248Arg) | Pathogenic |
|  |  | yes |  | p.(Gly248Arg) | Pathogenic |
| c.946C>T p.(Arg316Cys) | VUS | yes |  |  |  |
|  |  | yes |  | p.(Gly341Arg) | Pathogenic |
|  |  | yes |  | p.(Gly341Arg) | Pathogenic |
|  |  | yes |  | p.(Arg401Cys) | Pathogenic |
|  |  | yes |  | p.(Arg401His) | Pathogenic |
|  |  |  | yes | p.(Tyr522Ser) | Pathogenic |
|  |  |  | yes | p.(Tyr522Cys) | Likely pathogenic |
| c.1589G>A p.(Arg530His) | Likely Pathogenic | yes |  |  |  |
| c.1598G>A p.(Arg533His) | VUS | yes |  |  |  |
|  |  | yes |  | p.(Arg533Cys) | Pathogenic |
|  |  |  |  | p.(Phe539Leu) | Likely pathogenic |
|  |  | yes |  | p.(Arg552Trp) | Pathogenic |
| c.1762C>T p.(Leu588Phe) | VUS |  |  |  |  |
| c.1840C>T p.(Arg614Cys) | Pathogenic | yes |  | p.(Arg614Cys) | Pathogenic |
| c.1841G>T p.(Arg614Leu) | Pathogenic | yes |  | p.(Arg614Leu) | Pathogenic |
|  |  |  | yes | p.(Ser1728Phe) | Likely pathogenic |
| c.6385G>A p.(Asp2129Asn) | VUS | yes |  |  |  |
|  |  | yes |  | p.(Arg2163Cys) | Likely pathogenic |
| c.6488G>A p.(Arg2163His) | Pathogenic | yes |  | p.(Arg2163His) | Pathogenic |
|  |  | yes |  | p.(Arg2163Pro) | Likely pathogenic |
| c.6502G>A p.(Val2168Met) | Pathogenic |  |  | p.(Val2168Met) | Pathogenic |
|  |  | yes |  | p.(His2204Gln) | Likely pathogenic |
| c.6617C>T p.(Thr2206Met) | Pathogenic |  |  | p.(Thr2206Met) | Pathogenic |
|  |  | yes | yes | p.(Thr2206Arg) | Pathogenic |
|  |  |  |  | p.(Val2210Phe) | Likely pathogenic |
| c.6742C>T p.(Arg2248Cys) | VUS | yes |  |  |  |
| c.6863T>C p.(Leu2288Ser) | VUS |  | yes |  |  |
|  |  | yes |  | p.(Arg2336His) | Pathogenic |
| c.7035C>A p.(Ser2345Arg) | Likely pathogenic | yes | yes |  |  |
| c.7042_7044del p.(Glu2348del) | Likely Pathogenic | yes |  | p.(Glu2348del) | Pathogenic |
| c.7048G>A p.(Ala2350Thr) | Pathogenic |  | yes | p.(Ala2350Thr) | Pathogenic |
| c.7063C>T p.(Arg2355Trp) | Pathogenic | yes |  | p.(Arg2355Trp) | Pathogenic |
| c.7087T>C p.(Cys2363Arg) | VUS | yes |  |  |  |
|  |  |  |  | p.(Phe2364Val) | Likely pathogenic |
|  |  |  |  | p.(Gly2375Ala) | Pathogenic |
| c.7210G>A p.(Glu2404Lys) | VUS | yes |  |  |  |
| c.7268T>A p.(Met2423Lys) | VUS | yes |  |  |  |
|  |  |  | yes | p.(Ala2428Thr) | Pathogenic |
|  |  | yes | yes | p.(Asp2431Tyr) | Pathogenic |
| c.7300G>A p.(Gly2434Arg) | Pathogenic | yes |  | p.(Gly2434Arg) | Pathogenic |
|  |  | yes |  | p.(Arg2435His) | Pathogenic |
|  |  | yes |  | p.(Arg2435Leu) | Likely pathogenic |
|  |  | yes |  | p.(Ala2437Val) | Likely pathogenic |
|  |  | yes |  | p.(Arg2452Trp) | Pathogenic |
|  |  | yes |  | p.(Arg2454Cys) | Pathogenic |
| c.7361G>A p.(Arg2454His) | Pathogenic | yes |  | p.(Arg2454His) | Pathogenic |
| c.7373G>A p.(Arg2458His) | Pathogenic | yes |  | p.(Arg2458His) | Pathogenic |
|  |  | yes |  | p.(Arg2458Cys) | Pathogenic |
| c.7523G>A p.(Arg2508His) | Likely Pathogenic | yes |  | p.(Arg2508His) | Pathogenic |
|  |  | yes |  | p.(Arg2508Cys) | Pathogenic |
| c.8026C>T p.(Arg2676Trp) | Likely Pathogenic | yes |  | p.(Arg2676Trp) | Likely pathogenic |
|  |  | yes |  | p.(Glu3104Lys) | Pathogenic |
| c.10648C>T p.(Arg3550Trp) | VUS | yes |  |  |  |
|  |  | yes |  | p.(Arg3772Gln) | Likely pathogenic |
|  |  | yes |  | p.(Arg3903Gln) | Likely pathogenic |
|  |  |  |  | p.(Gly3990Val) | Pathogenic |
| c.12533G>T p.(Gly4178Val) | VUS |  |  | p.(Gly4178Val) | Likely pathogenic |
|  |  |  |  | p.(Val4234Leu) | Likely pathogenic |
|  |  | yes |  | p.(Arg4737Trp) | Likely pathogenic |
|  |  | yes |  | p.(Arg4737Gln) | Likely pathogenic |
|  |  |  |  | p.(Leu4824Pro) | Likely pathogenic |
|  |  |  | yes | p.(Thr4826Ile) | Pathogenic |
|  |  | yes | yes | p.(His4833Tyr) | Pathogenic |
|  |  |  |  | p.(Leu4838Val) | Pathogenic |
|  |  |  |  | p.(Val4849Ile) | Pathogenic |
|  |  | yes |  | p.(Arg4861His) | Pathogenic |
|  |  | yes |  | p.(Lys4876Arg) | Likely pathogenic |
|  |  |  |  | p.(Pro4973Leu) | Likely pathogenic |
